# Body image of people over 50 in Spain measured using the BSQ test

**DOI:** 10.1186/s13104-020-4913-9

**Published:** 2020-01-30

**Authors:** Roberto Sánchez-Cabrero, Fernando Martínez-López, Reyna Guadalupe Euán-Ramírez

**Affiliations:** Alfonso X the Wise University, Madrid, Spain

**Keywords:** Body image, Body satisfaction, Maturity, Old age, Elderly

## Abstract

**Objectives:**

To show the body image of people over 50 in Spain using the *Body Shape Questionnaire test* (BSQ), taking into account attribute variables of great interest such as age, gender, sentimental status, habitat (rural or urban) and the season of the year in which the test is done (winter or summer).

**Data description:**

The results obtained show the current state of the body image of 176 people in the process of ageing in Spain. The data collected from the participants are organised taking into account attribute variables of significant impact on body image such as age, gender, having a stable partner, habitat (rural or urban) and the season of the year in which the test is done (winter or summer). These data are especially useful to show how body image changes over the time, depending on the different attributes and according to diverse emotional and social situations. They can be used in studies on body image, eating disorders or studies that assess the importance of physical appearance in someone’s self-esteem regardless of age group, geographic area or personal emotional circumstances.

## Objective

Our main objective was to measure the body image of people over 50 in Spain using the *Body Shape Questionnaire* (BSQ) by Cooper, Taylor, Cooper and Fairburn [1], adapted to Spanish and validated according to the Spanish population by Raich et al. [2], as it is the most appropriate current test for this task due to its adequacy to the aging population, given its simplicity, acceptance and validity among the current international scientific community [3].

Different attribute variables of great interest, such as age [4–7], gender [4, 6, 8], whether having a stable partner [9], habitat [10] (rural or urban) and season of the year when the test was done [11, 12] (winter or summer), were taken into account when collecting the anonymous measurement data, in order to use them later as part of the research conducted by Sánchez-Cabrero et al. [3], in which the relationship between body satisfaction, as an affective component of body image, measured through the BSQ test, and vital satisfaction in ageing or elderly people was evaluated.

In order to carry out the field study that provided these data, several Spanish locations, both urban and rural (with less than 1000 inhabitants), were visited at different times of the year (gathering information in warm and cold seasons) and an in situ paper test was made. At the same time, along with the completion of the BSQ test, each participant anonymously attached their information regarding their age, gender and relationship status. In addition, the researcher who collected the data noted whether the measurement had taken place in an urban or rural area and also took note of the date of measurement, distinguishing between warm and cold seasons of the year.

## Data description

The data described here are quantitative; they were obtained from a single measurement individually made for each participant in a cross-sectional field study. Each participant completed the BSQ questionnaire individually with pen and paper. The participants were all recruited via the Spanish Red Cross in the North-West of Spain, which is one of the most affected areas by aging population problems. Sampling was done by clusters, having a total of 10 groups of people over 50 years old that were participating in social programs of the Spanish Red Cross. Half of the clusters came from rural places (localities with less than 1000 inhabitants) and the other half from urban areas. All of them were Caucasian (this Spanish region is not racially diverse). The measurements were collected at two different times of the year: summer (average daily temperature exceeding 20 °C) and winter (average daily temperature below 10 °C). These data show the results obtained through the replies given to the BSQ test [1], which measures body satisfaction as an evaluating factor of body image [13–15], by 176 people over 50 in Spain with different attribute profiles regarding age, gender, relationship status, habitat and season of the year in which the measurement took place, as shown in Data file 1 and 2, in Data set 1.

The BSQ test consists of 34 items that make the participant think about specific social and psychological situations connected to their concern about their own physical appearance, with a 6-point Likert scale, and measures his or her body satisfaction in the opposite way (the lower the score, the higher the body satisfaction). The BSQ gives a single score resulting from the sum of the points obtained in its 34 items [2].

Both the result obtained with the BSQ test and the age variable are reflected as discrete quantitative variables in integer values (see Data file 8). The rest of the variables are considered as dichotomous nominal variables with two conditions: gender (male or female), sentimental status (with or without a stable partner), habitat (rural or urban) and season of the year (warm or cold), as can be seen in the Data files 3–8.

Once all anonymous written responses to the BSQ test had been compiled, along with their data regarding the measurement variables, the final score and result of each participant were obtained, together with the concretion of the rest of the attribute variables, all this information was transferred to a data file in *.csv* format, for conversion into Microsoft Access format and their subsequent transformation into *.sav* format to manage the data with SPSS Statistical Software.

The data described here correspond directly to the results obtained by the study participants when completing the BSQ test and answering the questions of the researchers, without any filter or interpretation by the evaluators who collected the data.

The data collected show a specific score regarding body satisfaction obtained by the participants in the study, which clearly reflects a certain level of concern for one’s own physical appearance or body image according to the scales pre-established by the authors of the BSQ test [1]. The ‘*Satisfaction level*’ variable uses the levels established by Cooper et al. [1, 2] to place each participant in a category that matches with a predefined concern for the physical aspect (nonexistent, low, moderate and extreme), as shown in the Data file 9. These data show the importance that body image can have for people with the attribute characteristics taken into consideration for the study [16–18], as can be seen in Data files 3–8 (Table 1).

**Table 1:** Overview of data files/data sets

Label	Name of data file/data set	File types (file extension)	Data repository and identifier (DOI or accession number)
Data file 1	BSQ-RSC-2	SPSS files (.sav)	10.5061/dryad.vx0k6djmp
Data file 2	BSQ_freq.xlsx	MS Excel files (.xlsx)	10.5061/dryad.vx0k6djmp
Data file 3	BMC1300.tif	Images (.tif)	10.5061/dryad.vx0k6djmp
Data file 4	BMC2300.tif	Images (.tif)	10.5061/dryad.vx0k6djmp
Data file 5	BMC3300.tif	Images (.tif)	10.5061/dryad.vx0k6djmp
Data file 6	BMC4300.tif	Images (.tif)	10.5061/dryad.vx0k6djmp
Data file 7	BMC5300.tif	Images (.tif)	10.5061/dryad.vx0k6djmp
Data file 8	BMC6300.tif	Images (.tif)	10.5061/dryad.vx0k6djmp
Data file 9	BMC7300.tif	Images (.tif)	10.5061/dryad.vx0k6djmp

## Limitations

- Some of the items in the BSQ test are intended for much younger people’s way of thinking, therefore the older participants may feel confused when taking the test.
- The BSQ test focuses more on female concerns, so men may find themselves confused when completing the test.
- The categories pre-established by the original authors of the BSQ test to assess the concern for someone’s physical appearance are 30 years old, hence an update would be recommended.
- The ‘*Habitat*’ variable is less relevant in the globalised context in which we live today. However, for the older group of people, the social environment is still exclusively identified with the geographical and social environment of coexistence.
- The ‘*Sentimental status*’ variable could be further contextualised by differentiating, for example, between single, divorced or widowed people. However, this differentiation makes it difficult to evaluate the regulatory role that the fact of having a couple can have on being satisfied with the appearance of one’s own body.

## Data Availability

The data described in this Data note can be freely and openly accessed on *Dryad repository:* 10.5061/dryad.vx0k6djmp [19]. Please see Table 1 and reference list for details and links to the data.

## Abbreviations

BSQ: *Body Shape Questionnaire*
SPSS: Statistical Package for the Social Sciences
